# Building heterogeneous nanostructures for photocatalytic ammonia decomposition

**DOI:** 10.1039/d0na00161a

**Published:** 2020-07-10

**Authors:** Shijie Zhang, Zuoli He, Xuan Li, Jing Zhang, Qianhao Zang, Shuguang Wang

**Affiliations:** Shandong Key Laboratory of Water Pollution Control and Resource Reuse, School of Environmental Science and Engineering, Shandong University Qingdao 266237 China zlhe@sdu.edu.cn wsg@sdu.edu.cn; School of Materials Science and Engineering, Jiangsu University of Science and Technology Zhenjiang 212003 China

## Abstract

Ammonia is an important chemical for human beings that is used in the synthesis of chemical fertilizers and products; meanwhile, it is also a hazardous compound which causes undesirable odors, several diseases, and environmental problems. Therefore, there is an urgent need to control and remove ammonia pollutants from water, air and soil. Hence, clean processes using photocatalysis to convert ammonia into H_2_ and N_2_ have been an important research topic in recent years. To date, only some metal-loaded common photocatalysts, such as TiO_2_, ZnO, C_3_N_4_, graphene and other carbon-based materials together with their hybrid materials, have been reported as active photocatalysts for the decomposition of aqueous ammonia solutions. In this review, we summarize the recent advances in heterogeneous nanostructures for photocatalytic ammonia decomposition. Particular emphasis is also given to metal-loading along with the resulting heterojunctions. Furthermore, the recent efforts toward the development of heterogeneous nanostructures for photocatalytic ammonia decomposition in this direction are discussed and appraised. Finally, perspectives and future opportunities regarding the challenges and future directions in the area of heterogeneous photocatalysts for ammonia decomposition are also provided.

## Abstract 2

In this review, we summarize the recent advantages of heterogeneous nanostructures for photocatalytic ammonia decomposition.

## Graphical abstract

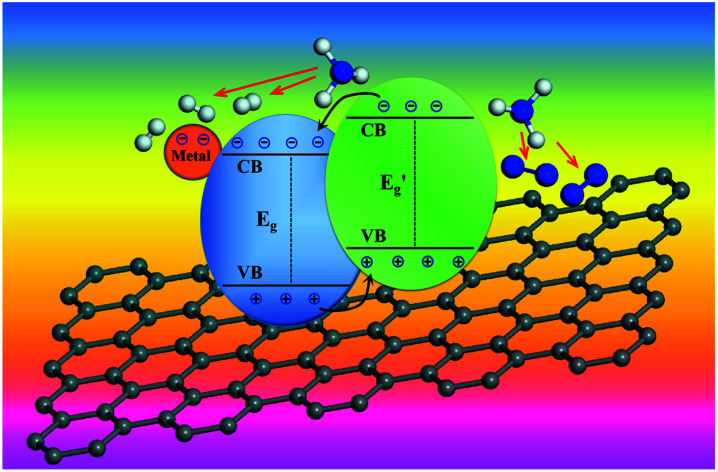

## Introduction

In the past few decades, unavoidable consequences of fossil fuels and their excessive use have caused numerous negative effects on human health as well as on the environment, especially in developing countries.^1–3^ Therefore, there is a strong need to establish eco-friendly and sustainable alternatives to address environmental pollution while maintaining sustainable development of the global economy.^4,5^ Excess nitrogen compounds have been discharged from many types of industrial operations, such as chemical engineering and power plants as well as livestock industry facilities.^6–11^ For example, ammonia is mainly produced from the microbial decomposition of nitrogen-containing organic substances at very low oxygen concentrations and the decomposition of urea and nitrogen compounds in wastewater treatment processes. As a hazardous compound, it causes undesirable odors, several diseases and environmental problems; thus, there is an urgent need to control and remove ammonia pollutants from water, air and soil.^12,13^ It should be noted that ammonia is also a promising hydrogen carrier because it possesses an excellent energy density and storage capacity of H_2_ of 17.6 wt% (compared to other storage materials such as cyclohexane, ethanol and liquefied petroleum gas).^14,15^ Hydrogen production from ammonia decomposition is an eco-friendly and sustainable alternative to transform ammonia pollutants into resources.^16–18^ As shown in eqn (1)–(3), hydrogen production from ammonia decomposition is an uphill reaction from the thermodynamics point of view and requires energy for the reactions, and the Gibbs free energy of ammonia decomposition is far lower than that of water splitting; thus, a photocatalyst for water splitting can convert photon energy into chemical energy through this reaction.^19,20^1
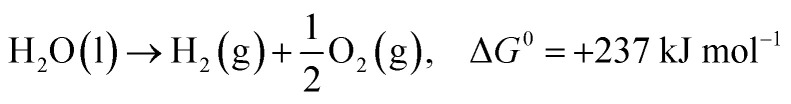
2
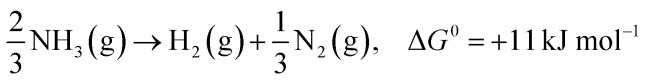
3
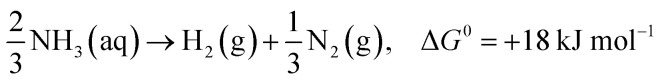


In the last ten years, many researchers working on the conversion of NH_3_ into non-toxic H_2_ and N_2_ (an uphill reaction, Δ*G* = 11 or 18 kJ mol^−1^) have made their best efforts to improve catalytic systems to better mimic CO_*x*_-free hydrogen production performance.^21^ Therefore, this eco-friendly artificial synthetic reaction will be an important research topic. To date, three main catalytic approaches, namely thermal-catalysis,^22–24^ electro-catalysis^25,26^ and photo-catalysis,^27–29^ have been utilized for ammonia decomposition. Pertinent studies have proved that solar energy is an effective alternative among the various environmentally friendly energies, which is attributed to its great potential in electricity generation, chemical fuel production and pollutant degradation. Fujishima and Honda discovered the phenomenon of photocatalytic water splitting on titanium dioxide (TiO_2_) electrodes in 1972;^30^ this triggered the development of semiconductor photocatalysts for a wide range of environmental and energy applications, including degradation of organic pollutants, water splitting, nitrogen fixation, hydrogen production from ammonia, and carbon dioxide reduction. The efficiency of photocatalysis is related to its efficiency in five main steps during the reactions: (i) light absorption, (ii) formation of photogenerated electron–hole pairs, (iii) migration and separation of photogenerated electron–hole pairs, (iv) adsorption of reactants and desorption of products, and (v) occurrence of redox reactions on the semiconductor surface.^31,32^ Building heterogeneous nanostructures has been proved to enhance photocatalytic activity by increasing some of these five efficiencies; this often involves defect engineering, surface engineering, interface engineering and heterojunction engineering.

**Fig. 1:**
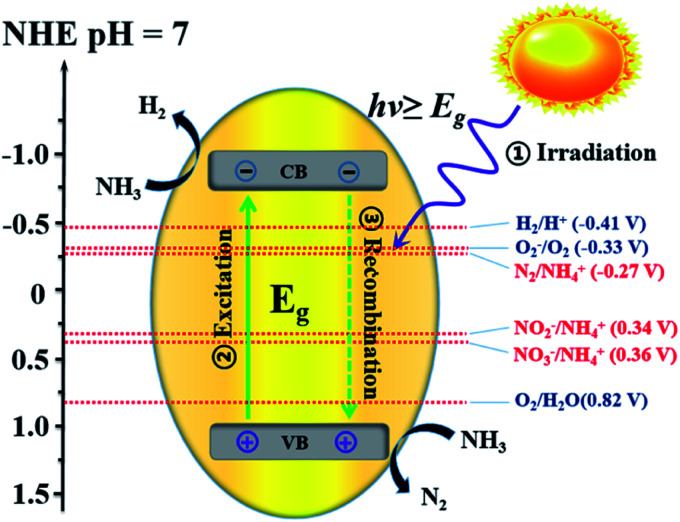
Illustration of the process of photocatalytic ammonia decomposition.

For uphill reactions such as water splitting, hydrogen production from ammonia, and carbon dioxide reduction, the slow surface multi-electron reaction kinetics leads to inevitable accumulation of photogenerated electrons and holes on the surface of photocatalysts, which accelerates the unexpected charge carrier recombination and the photocorrosion process of the photocatalysts themselves and consequently reduces their photoactivity.^33^ Photocatalytic decomposition of ammonia using sunlight, as an artificial uphill photosynthetic reaction, often takes place in alkaline conditions. When the irradiating light has higher energy than the band gap of the photocatalyst, electron–hole pairs will generally form (eqn (4)). The generated holes (h^+^) have been demonstrated to be powerful oxidants, whereas the conduction band electrons (e^−^) can act as strong reductants to reduce O_2_ for the formation of hydroxyl radicals (˙OH). The holes or ˙OH for the oxidation of ammonia to N_2_ or NO_*x*_ are provided by the reactions shown in eqn (5)–(10).^34^ H_2_ production by the decomposition of an aqueous ammonia solution is an uphill reaction, as mentioned above. Thus, an uphill reaction to convert photon energy into chemical energy will be achieved using a heterogeneous photocatalyst driven by one-step photoexcitation. As shown in Fig. 1, electron/hole pairs can be generated by photons with higher energy than the corresponding band-gap energy of the photocatalyst; some of these electron/hole pairs can migrate to the surface or defects and initiate a series of chemical reactions with the adsorbed species on the surface of the catalyst, resulting in the degradation of ammonia. From the thermodynamic viewpoint, only when the ammonia reduction and oxidation potentials lie between the conduction band (CB) and the valence band (VB) potentials can such reactions be driven by photogenerated electrons and holes. During this process, different nitrogen-containing products (*i.e.*, N_2_, NO_2_^−^, NO_3_^−^, and NO_*x*_) can be produced due to their similar redox potentials; the potentials of these typical reactions are given in Fig. 1.^35^ Ammonia can be decomposed into different amounts of various products under diverse reaction conditions (*i.e.*, pH, temperature, initial ammonia or O_2_ concentration, trapping agent, sacrificial agent). To achieve the photocatalytic degradation of ammonia, the photogenerated electrons and holes on the surface of a semiconductor should have suitable reduction and oxidation ability to react with the adsorbed species (*i.e.*, O_2_, NH_4_^+^, NO_2_^−^, NO_3_) on the surface of the catalyst and generate free radicals or different products.

4 Photocatalyst + *hν* → e^−^ + h^+^

5 OH^−^ + h^+^ → OH˙

6 O_2_ + e^−^ → O_2_^−^˙

7 O_2_ + 2e^−^ + 2H^+^ → H_2_O_2_

8 2O_2_^−^˙ + 2H^+^ → H_2_O_2_ + O_2_

9 H_2_O_2_ + e^−^ → OH^−^ + OH˙

10 H_2_O_2_ + O_2_^−^˙ → OH^−^ + OH˙ + O_2_

Moreover, photocatalytic decomposition of ammonia usually stops under acidic conditions, which indicates that H^+^ ions inhibit the conversion of ammonia to NH_2_ free radicals. When the pH gradually increases, ammonia may react with surrounding oxygen to produce NO_2_^−^, NO_3_^−^ and other nitrogen oxides (eqn (11)–(13)), which is detrimental to hydrogen production.^36^

11 NH_3_ + 3O_2_ → 2NO_2_^−^ + 2H^+^ + 2H_2_O, Δ*G*^0^ = +11 kJ mol^−1^

12 2NO_2_^−^ + O_2_ → NO_3_^−^, Δ*G*^0^ = +11 kJ mol^−1^

13 NH_3_ + 2O_2_ → NO_3_^−^ + H^+^ + H_2_O, Δ*G*^0^ = +11 kJ mol^−1^

To date, only some metal-loaded common photocatalysts, such as TiO_2_, ZnO, C_3_N_4_, graphene and other carbon-based materials, together with their hybrid materials have been reported as active photocatalysts for the decomposition of an aqueous ammonia solution into H_2_ and N_2_, as presented in Table 1.^19,25,37,38^ In this review, particular emphasis will first be given to metal-loading along with the resulting heterojunctions; then, the recent developments of TiO_2_, ZnO, C_3_N_4_, graphene and other carbon-based materials in the application of ammonia decomposition will be respectively described. Finally, some concluding remarks and perspectives on the challenges and opportunities for exploring efficient heterogeneous photocatalysts for ammonia decomposition are presented. We hope that this review will assist and inspire further development of efficient heterogeneous materials for ammonia decomposition.

**Table 1:** Heterogeneous nanostructures for the photocatalytic degradation and decomposition of ammonia

Photocatalyst	Synthesis method	Band gap	Light source	Illumination	Initial ammonia concentration	Maximum decomposition ability	Ref.
Pt–TiO_2_ (0.5 wt% Pt)	Photochemical reduction	—	450 W high pressure Hg lamp	298 K, 24 h	5 mM	Over 95%	36
Ni/TiO_2_ (0.5 wt% Ni)	Impregnation method	—	500 W Xe lamp	3 h	5 mL, 0.59 mol L^−1^	131.7 μmol H_2_ per g-catalyst	39
Ce-doped TiO_2_ (0.6 wt% Ce)	Sol–gel method	3.14 eV	8 W Hg pen-ray lamp	10 h	100 mL, 0.8274 g L^−1^	810 mmol H_2_ per g-catalyst	40
Ce-doped TiO_2_ (1.2 wt% Ce)	Sol–gel method	3.00 eV	8 W Hg pen-ray lamp	10 h	100 mL, 0.8274 g L^−1^	1060 mmol H_2_ per g-catalyst	40
Ce-doped TiO_2_ (1.4 wt% Ce)	Sol–gel method	2.97 eV	8 W Hg pen-ray lamp	10 h	100 mL, 0.8274 g L^−1^	1010 mmol H_2_ per g-catalyst	40
Pt/Fe–TiO_2_ (0.5 wt% Pt; 1.0 wt% Fe)	Impregnation method	—	500 W Xe lamp	2 h	5 mL, 0.59 mol L^−1^	27 μmol H_2_ per g-catalyst	41
Light expanded clay aggregate (LECA)-TiO_2_ nanoparticles	Thermal annealing	—	80 W medium-pressure Hg lamp	3 h	0.05 M, 750 mL	85%	42
ZnO/Ag (molar ratio 2 : 1)	One-pot method	—	300 W Xe lamp	298 K, 2.5 h	1.5 mg L^−1^	*Circa* 90%	43
ZnO	Thermal annealing	3.26 eV	8 W Hg pen-ray lamp	8 h	0.8274 g L^−1^	1060 μmol H_2_ per g-catalyst	19
ZnO	Precipitation reaction + thermal annealing	3.24 eV	8 W Hg pen-ray lamp	8 h	0.8274 g L^−1^	670 μmol H_2_ per g-catalyst	19
ZnO	Precipitation reaction + UV irradiation + thermal annealing	3.23 eV	8 W Hg pen-ray lamp	8 h	0.8274 g L^−1^	860 μmol H_2_ per g-catalyst	19
TiO_2_–ZnO (molar ratio 1 : 2)/LECA (light expanded clay aggregate)	Impregnation method	3.22 eV	125 W high pressure Hg lamp	293 K, 3 h	400 mg L^−1^	95.2%	44
ZnO/oak charcoal	Impregnation method	—	125 W Hg lamp	293 K, 4 h	153.9 mg L^−1^	About 80%	34
N–C@TiO_2_ (nitrogen-doped carbon framework)	hydrothermal method	—	25 W UV lamp	295 ± 1.5 K, 5 min	100 μL aqueous ammonia (30%)	100%	45
MoS_2_@TiO_2_ CNBs (carbon nanobelts)	Electrospinning followed by hydrothermal reaction method	—	25 W UV lamp	295 ± 1.5 K, 7 min	100 μL aqueous ammonia (30%)	91%	46
MoS_2_/N-doped graphene (5% of MoS_2_ mass)	Hydrothermal method	0.80 eV	300 W UV-visible lamp	298 ± 2 K, 8 h	100.0 mg L^−1^	99.6%	47
Nitrogen-doped rGO/TiO_2_ nanowires (NWs) membranes	Modified Hummers method + adsorption method	—	8 W Hg pen-ray lamp	12 h	0.883 g L^−1^	208 μmol h^−1^ g^−1^	48
SL g (single layer graphitic)-C_3_N_4_	Thermal annealing + hydrothermal method	3.0 eV	Xe lamp (195 mW cm^−2^)	298 K, 6 h	1.5 mg L^−1^	Over 80%	35
GQDs (graphene quantum dots)/CN (g-C_3_N_4_) (0.5 wt% GQDs)	Thermal annealing + Hummer's method + hydrothermal method	2.7 eV	150 W Xe arc lamp	7 h	1.5 mg L^−1^	90%	49

## Building heterogeneous nanostructures

**Fig. 2:**
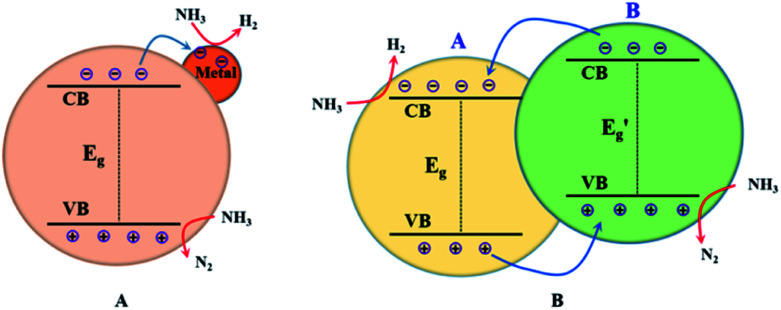
Illustrations of metal-loading (A) and heterojunction building (B) for enhanced photocatalytic ammonia decomposition performance.

As mentioned above, building heterogeneous nanostructures has been proved to enhance photocatalytic performance by increasing some of the five efficiencies during photocatalysis reactions; this often involves defect engineering, surface engineering, interface engineering and heterojunction engineering. For ammonia decomposition, heterogeneous photocatalysts are often obtained by metal-loading and constructing heterojunctions with semiconductor photocatalysts for use in water splitting. As shown in Fig. 2, metal loading will introduce defect positions, high-speed charge transfer paths to increase the efficiencies of migration and separation of the photogenerated electron–hole pairs, adsorption of reactants and desorption of products, and occurrence of redox reactions on the semiconductor surface.^50–57^ Heterojunctions improve the activity of photocatalysts because of their suitable structures, which improve charge separation and transfer, promote optical absorption, optimize the bandgap position, and enhance the spatial separation of electron–hole pairs; this can increase some of the five efficiencies during photocatalysis, namely light absorption, formation of photogenerated electron–hole pairs, migration and separation of the photogenerated electron–hole pairs, adsorption of reactants and desorption of products, and occurrence of redox reactions on the semiconductor surface.^50–52^

**Fig. 3:**
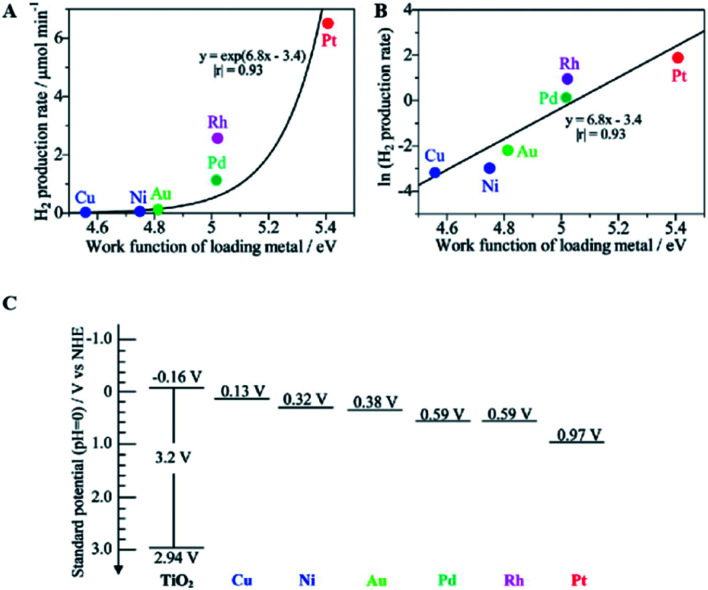
Relationship between the hydrogen production rate over the M(0.1)/TiO_2_ samples and the work function of the corresponding bulk metal (A), the logarithmic plot (B), and the standard potentials for the band edge positions of titanium oxide and the Fermi level of each loading metal (C). Reprinted with permission from ref. 62. Copyright 2012 American Chemical Society.

Taking metal-loaded TiO_2_ as an example to more deeply describe the advances in photocatalytic ammonia decomposition, metal loading can inhibit the recombination of electrons and holes on the surface of TiO_2_ to extend the carrier lifetimes by introducing defect positions or high-speed charge transfer paths.^36,41,58–62^ Furthermore, it can create active sites to increase the number of reaction locations and the adsorption capabilities of the reactants. Overall, metal loading will lead to enhanced photocatalytic performance and provide better chemical and environmental stability. Metal-loaded photocatalysts show higher activities than bare TiO_2_ to produce nitrogen and hydrogen from ammonia with an almost stoichiometric product ratio.^62^ As shown in Fig. 3A, among these metal-loaded TiO_2_ samples, metals with larger work functions are effective for gaseous ammonia decomposition. The most suitable cocatalyst for hydrogen evolution is Pt, which exhibits a high work function, low Fermi level and strong electron extracting capacity. In addition, it can be easily loaded by photo-deposition from a molecular precursor. In addition, the work function of the bulk metal and the logarithm of the hydrogen production rate have an almost linear relationship, as shown in Fig. 3B; this indicates that the photocatalytic hydrogen production activity in ammonia decomposition is mainly derived from the charge separation ability of the photogenerated hole and electron pairs rather than the original catalytic properties of the loaded metals for hydrogen production. In other words, the loaded metal with a larger work function, *i.e.*, a lower Fermi level, will more easily accept the excited electrons from the conduction band (−0.16 V *vs.* NHE, Fig. 3C) to promote H_2_ production for ammonia decomposition. Although metal loading can effectively inhibit the recombination of electrons and holes on the catalyst surface, overloading of metal can also cause more recombination centers to appear and can affect the performance of the photocatalyst. Therefore, the optimal weight ratio of the metal loading to the main photocatalyst is mostly lower than 3 wt%.

In general, a heterojunction photocatalyst contains an interface between two different semiconductors with unequal band structures, which can result in suitable band alignments for photocatalytic reactions. For type-II heterojunction photocatalysts (see Fig. 2B), the CB and the VB levels of semiconductor B are higher than the corresponding levels of semiconductor A. Thus, the photogenerated electrons will transfer to semiconductor A while the photogenerated holes will migrate to semiconductor B, which will result in spatial separation of the electron–hole pairs.^63^ The redox ability of these heterojunction photocatalysts will also be reduced because the reduction reaction and the oxidation reaction take place on semiconductor A with lower reduction potential and on semiconductor B with lower oxidation potential, respectively.^64^ Generally, this type of heterojunction photocatalyst exhibits a smaller band-gap, good electron–hole separation, a wide light-absorption range, fast mass transfer and a larger surface area; this will increase the efficiency of some processes during photocatalysis, such as light absorption, formation of photogenerated electron–hole pairs, and migration and separation of the photogenerated electron–hole pairs. Additionally, metal-loading and heterojunction building have been applied together to design highly efficient ammonia decomposition photocatalysts. In the following sections, we provide a comprehensive overview of the ammonia decomposition photocatalysts, summarizing recent related studies in this area.

## Metal-loaded TiO_2_ and its heterojunction photocatalysts

TiO_2_ is one of the most common photocatalysts; it has good performance in many photocatalytic applications, such as water splitting, hydrogen production, carbon dioxide reduction, nitrogen oxide reduction, nitrogen fixation, pollutant degradation, organic reactions, and ammonia synthesis and decomposition.^31,42,65–74^ Three crystal types of TiO_2_, including anatase type, rutile type and plate titanium type, exist in nature, with bandgap widths of 3.0, 3.2 and 3.25 eV, respectively.^75^ Bare TiO_2_ shows lower photocatalytic activity in ammonia decomposition due to the reactive energy barrier. Ammonia decomposition is an uphill reaction to convert photon energy into chemical energy; thus, it must be achieved by a heterogeneous photocatalyst driven by one-step photoexcitation. Additionally, the photocatalytic efficiency of TiO_2_ on the surface is relatively low due to the rapid recombination of photocarriers.^76^ Therefore, in some studies, TiO_2_ has been modified with loading metals and heterojunctions have been constructed with other semiconductors to overcome these limits.^45,77^

**Fig. 4:**
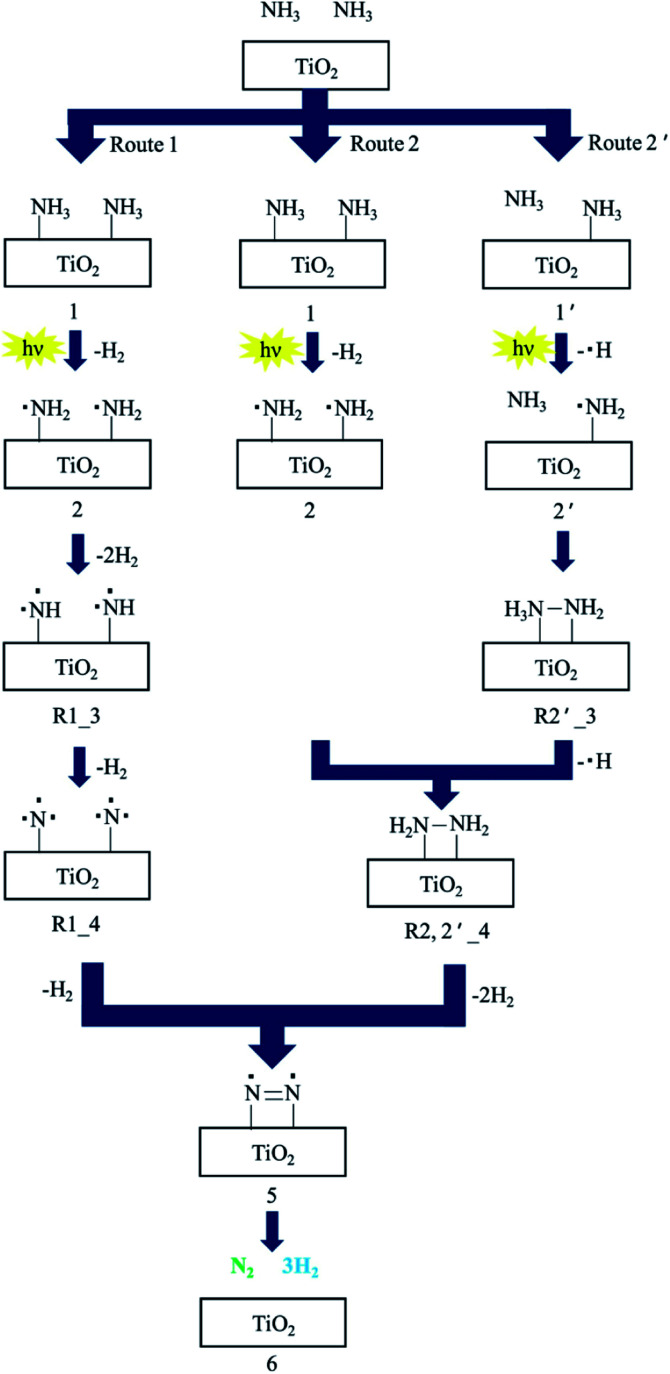
The suggested reaction mechanism for decomposition of NH_3_ to N_2_ and H_2_ over TiO_2_ photocatalysts. Reprinted from ref. 39, Copyright 2017, with permission from Elsevier.

Utsunomiya *et al.* compared the photocatalytic activities of ammonia decomposition of TiO_2_ loaded with various metals under UV irradiation at room temperature.^39^ As clearly shown in Table 2, Ni/TiO_2_ photocatalysts demonstrate the highest yields of H_2_. The NH_2_ radical was formed as a dominant intermediate during NH_3_ decomposition, which was confirmed by ESR measurements. H_2_ and H_2_N–NH_2_ as an intermediate were formed by the coupling of an NH_2_ radical and an NH_3_ molecule in the gas phase during the photodecomposition of NH_3_. XRD patterns proved that the crystal structure of Ni/TiO_2_ was stable, which further demonstrated that Ni has a good catalytic decomposition effect on ammonia. ESR measurements showed that NH_2_ radical is the dominant intermediate in the decomposition process. Ni^0^ was suggested to enhance the reaction pathways *via* H_2_N–NH_2_ because Ni^0^ was present in the catalyst that showed the highest catalytic activity. They also proposed three reaction pathways to investigate the mechanism of NH_3_ decomposition, as presented in Fig. 4. N_2_ and H_2_ were produced with the aid of an NH_2_ radical: route 1 involved the formation of NH radicals through extraction of one hydrogen atom from each of two NH_2_ radicals; route 2 involved the formation of NH_2_–NH_2_ by the coupling of adjacent NH_2_ radicals; and route 2′ involved the formation of NH_2_–NH_2_*via* formation of H_2_N–NH_3_. The activation energies for routes 1 and 2 were also calculated as 236 kcal mol^−1^ and 74.8 kcal mol^−1^, respectively, by density functional theory (DFT). Route 2 was found to be more energetically favorable than route 1. Possible reaction pathways by which N_2_ and H_2_ were formed through NH_2_–NH_2_ coupling were further split into route 2, involving the formation of H_2_N–NH_2_ by coupling of NH_2_ radicals; route 2′ involved the interaction of NH_2_ with one NH_3_ molecule in the gas phase. Their activation energies were estimated to be 74.4 kcal mol^−1^ and 59.2 kcal mol^−1^, respectively. Therefore, it is possible that NH_3_ decomposition proceeded by both routes 2 and 2′ *via* the formation of NH_2_–NH_2_.

**Table 2:** Comparison of the yields of H_2_ from NH_3_ decomposition in the presence of different M/TiO_2_ catalysts. Yield of H_2_ on NH_3_ photodecomposition over 0.5 wt% M/TiO_2_ (irradiation time: 3 h)^b^

Entry no.	Catalyst	Yield of H_2_ (μmol per g-cat.)
1	TiO_2_	8.7
2	V/TiO_2_	6.0
3	Cr/TiO_2_	7.0
4	Mn/TiO_2_	7.2
5	Fe/TiO_2_	6.7
6	Co/TiO_2_	6.3
7	Cu/TiO_2_	6.8
8	Ni/TiO_2_	131.7
9	Ni/TiO_2_^a^	8.4

a Without H_2_ reduction.

b Reprinted from ref. 39, Copyright 2017, with permission from Elsevier.

**Fig. 5:**
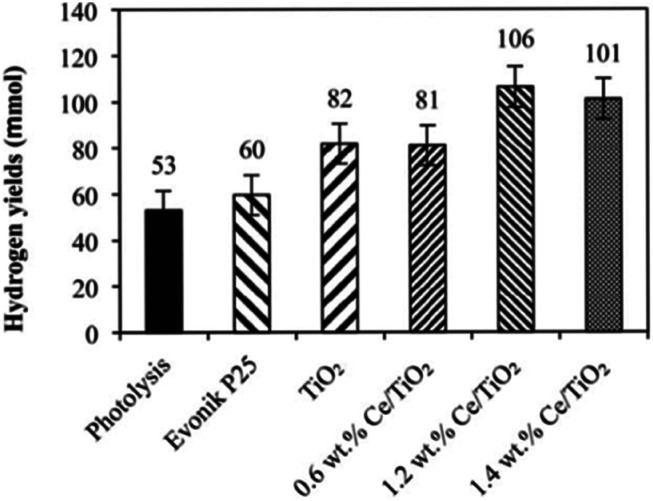
Hydrogen yields over each investigated photocatalyst and in the case of photolysis after 10 h irradiation. Conditions: 10 h of irradiation, 8 W Hg lamp (254 nm), 100 mL of NH_4_OH, 0.1 g of the photocatalyst. Reprinted from ref. 40. Copyright 2015, with permission from Elsevier.

The influence of Ce loading on the performance of photocatalytic ammonia decomposition was also investigated by Reli *et al.*^40^ Cerium-doped TiO_2_ photocatalysts with different contents (0–1.4 wt%) were prepared within reverse micelles of nonionic surfactant Triton X-114 in cyclohexane. The anatase crystallite sizes decreased with increasing amount of cerium ions in TiO_2_, which corresponded to the increase of the specific surface area of the catalysts. The addition of cerium led to a decrease of the absorption edge from 3.19 eV to 2.97 eV. NH_3_ was decomposed to H_2_ and N_2_ stoichiometrically and side reactions such as deep oxidation to NO^2−^ and NO^3−^ did not occur when the reaction was carried out in their stirred batch annular reactor. This metal modification of TiO_2_ influenced the photocatalytic hydrogen formation. The direct correlation between the work functions, band gap energies and hydrogen yields of the catalysts was investigated in this research, as shown in Fig. 5. The optimal Ce content in anatase TiO_2_ will maximally lower the bandgap energy while maintaining sufficient electron and hole potentials for the photocatalytic reaction.

The reaction mechanism of ammonia decomposition to nitrogen and hydrogen over platinum-loaded titanium oxide photocatalysts has been investigated through various reaction tests, as well as ESR and FT-IR spectroscopy.^62^ The pH of the reaction suspension can strongly affect both the oxidation rate and selectivity, which was confirmed by Shibuya *et al.*^36^ They investigated the influence of pH and pH adjustment conditions on the photocatalytic oxidation of aqueous ammonia with Pt–TiO_2_ under airflow. The oxidation of ammonia could not proceed under acidic conditions and could only proceed under alkaline conditions, suggesting that molecular ammonia reacts with photogenerated holes. Both the maximum oxidation rate and the maximum amount of ammonia adsorbed on the photocatalyst are observed at ≈pH 10; increasing the adsorbed capability of molecular ammonia is important to increase the oxidation rate. The decrease in pH due to the formation of nitrite and nitrate from the photo-oxidation of ammonia and the cessation of the reaction at pH values below 7 indicated that for the oxidation of ammonia, the pH must be maintained in the alkaline region during the reaction. Moreover, the amount of ammonia adsorbed when the pH was adjusted with NaOH (aq) was higher than that achieved using the Na_2_CO_3_–NaHCO_3_ buffer solution at the same pH because carbonate/hydrocarbonate ions prevent the adsorption of ammonia and the photogenerated holes are consumed by the oxidation of nitrite in the presence of the buffer. A high initial concentration of ammonia and pH adjustment to 10 with NaOH (aq) promotes the oxidation of ammonia with low selectivity for the undesirable products nitrite and nitrate. Performing the reaction with a high density of N-containing species on the photocatalyst surface favorably increases the selectivity for dinitrogen.

**Fig. 6:**
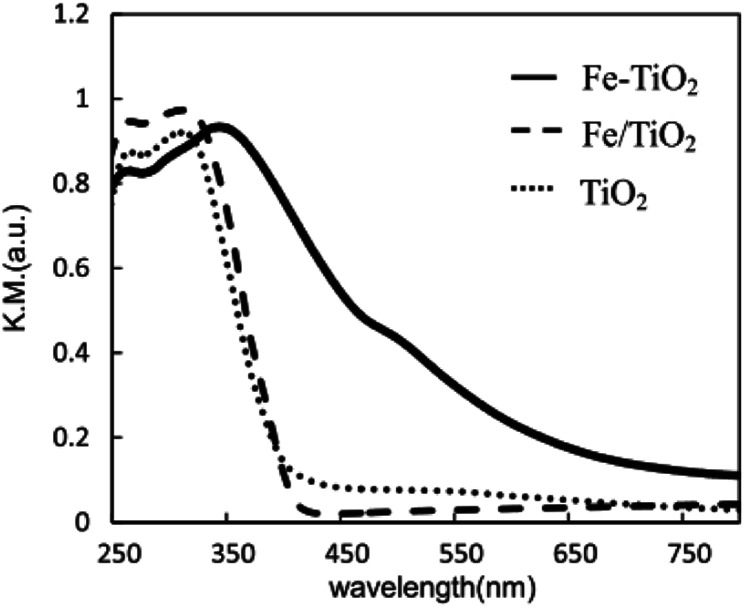
UV-vis-DR spectra of Fe–TiO_2_, Fe/TiO_2_ and TiO_2_ photocatalysts. Reprinted from ref. 41. Copyright 2015, with permission from Elsevier.

Obata *et al.* analyzed the performance of Pt-supported metal-doped TiO_2_ (Pt/M-TiO_2_, M: dopant element) photocatalysts on decomposing NH_3_ to H_2_ and N_2_ by ultraviolet radiation at room temperature (Table 3).^41^ Fe-doped TiO_2_ photocatalyst (Pt/Fe–TiO_2_) obtained the highest yield of H_2_ production in ammonia photodecomposition. The Fe^3+^ replaced the Ti^4+^ sites in TiO_2_ crystal without changing the structure of TiO_2_, and the absorption edge of TiO_2_ was shifted from the ultraviolet to the visible light region by Fe substitution, as shown in Fig. 6. The resulting Fe–TiO_2_ catalyst material allows the effective utilization of irradiation light owing to the presence of an Fe impurity band, thereby leading to higher activity of decomposing NH_3_ aqueous solution and more effective hydrogen production.

**Table 3:** The photocatalytic yields of H_2_ under various conditions: Pt loading, 0.5 wt%; Fe and Cr dopant, 1.0 wt%^a^

Sample no.	Catalyst	Reactants	Yield of H_2_ (μmol per g-catal.)
1	Pt/TiO_2_	H_2_O	0
2	Pt/TiO_2_	NH_3_ + H_2_O	18
3	Pt/Cr–TiO_2_	NH_3_ + H_2_O	14
4	Pt/Fe–TiO_2_	H_2_O	0
5	Pt/Fe–TiO_2_	NH_3_ + H_2_O	27
6	Pt/Fe–TiO_2_	NH_3_ + H_2_O	10

a Reprinted from ref. 41. Copyright 2014, with permission from Elsevier.

In addition, TiO_2_ nanoparticles supported on light expanded clay aggregate granules (LECA) showed enhanced photocatalytic decomposition of ammonia.^42^ More than 85% of the ammonia was removed within 300 min under the optimum calcination temperature of 550 °C and pH 11.0. The kinetics of this photocatalytic reaction followed a pseudo-first-order model. By using floated TiO_2_/LECA as the photocatalyst in an aqueous solution of NH_3_, ammonia was photodegraded into N_2_ and H_2_ gases, while NO_2_^−^ and NO_3_^−^ were formed at very low concentrations. Furthermore, they reported a novel hybrid structure of TiO_2_/ZnO/LECA for efficient removal of ammonia from synthetic wastewater,^44^ which will be further discussed in the following section.

## ZnO-based heterogeneous photocatalysts

Similar to TiO_2_, ZnO is one of the most widely used photocatalysts due to its high photocatalytic efficiency, low cost, and environmental sustainability.^78–80^ Its photocatalytic performance can be effectively improved by tailoring its crystallite size, surface area, and concentrations of active sites, oxygen defects, and facets.^43,81–84^ Moreover, metal-loaded ZnO and its heterojunctions have been used for photocatalytic decomposition of ammonia.

Different synthetic procedures lead to the formation of different defects (oxygen vacancies and oxygen excess defects) in the ZnO lattice, which is a factor influencing the photocatalytic hydrogen production efficiency of decomposition of ammonia solution under ultraviolet light of 254 nm.^19^ The highest yields of hydrogen were observed in ZnO prepared by thermal annealing of zinc acetate in air, as shown in Table 4. This photocatalyst possesses the lowest concentration of oxygen vacancies, causing efficient trapping of electrons. Consequently, a higher number of electrons in the conduction band was available for photocatalytic decomposition of ammonia.

**Table 4:** Physicochemical and optical properties of prepared photocatalysts and photocatalytic H_2_ generation relative to the commercial photocatalyst Evonik TiO_2_ P25^b^

Photocatalyst	Preparation method	*L*_c_ (nm)	*S*_BET_ (m^2^ g^−1^)	Band gap (eV)	H_2_ evolution^a^ (μmol g^−1^)	Rate of H_2_ production (μmol H_2_ per g per h)
ZnO (1)	Thermal annealing	36	15	3.26	1060	10.3
ZnO (2)	Precipitation reaction + thermal annealing	27	49	3.24	670	5.8
ZnO (3)	Precipitation reaction + UV irradiation + thermal annealing	15	43	3.23	860	6.8
Evonik TiO_2_ P25	Commercial	26	50	3.39	800	7.3

a Values depict photocatalytic activities after 8 h irradiation.

b Reprinted from ref. 19. Copyright 2015, with permission from Elsevier.

As mentioned above, metal loading or heterojunction building has been proved to be an efficient approach to enhance the photocatalytic activity for ammonia decomposition. Guo *et al.* synthesized a porous Ag-loaded ZnO heterogeneous photocatalyst by a one-pot method, and the interface contact and the electron transfer capacity between ZnO and other materials were proved to be enhanced.^43^ The photocatalytic activity for decomposition of ammonia was significantly improved after Ag modification and retained high efficiency in the stability experiment; this can be attributed to inhibition of the recombination process of photogenerated electrons and holes by interconversion between Ag^+^ and Ag^0^ at the surface of ZnO. In addition, the effective separation of the photogenerated carriers can generate more active groups, which can promote the degradation of ammonia or organic dyes.

**Fig. 7:**
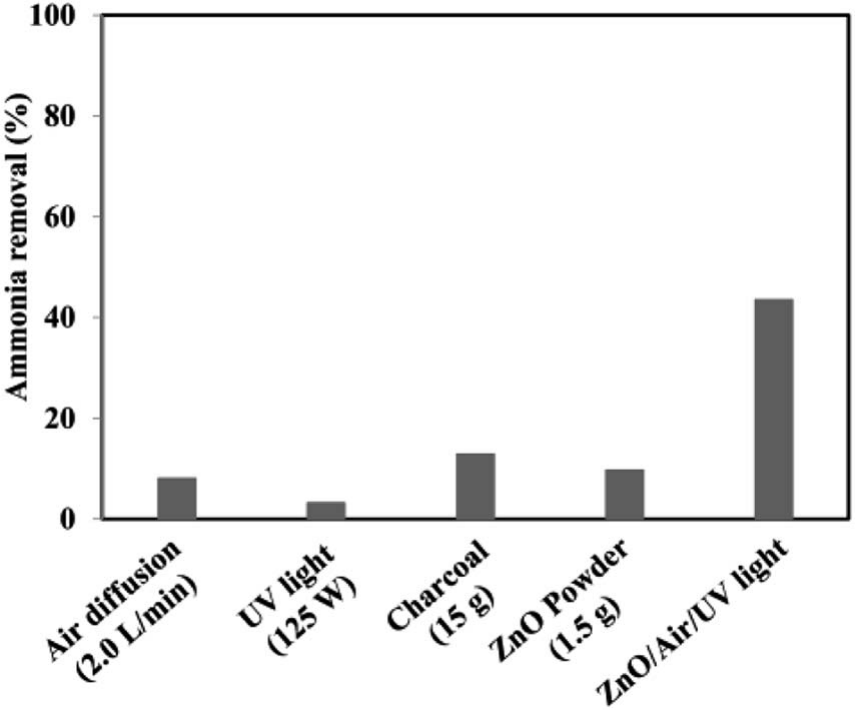
The results of reference tests to determine the proportion of adsorption and stripping as well as the effects of UV light and catalyst floating in the total removal of ammonia. Reprinted from ref. 34. Copyright 2017, with permission from Elsevier.

The experimental conditions of photocatalytic removal of ammonia from synthetic wastewater by a ZnO/oak charcoal photocatalyst were optimized using the Box–Behnken experimental design method.^34^ The contributions of different experimental conditions to the removal rate of ammonia were measured (Fig. 7), and the experimental data were fitted with a second-order polynomial regression model to derive the optimal experimental conditions, under which the maximum degradation efficiency of ammonia was about 80%. All three main variables of initial ammonia concentration, pH and catalyst dosage were effective in the ammonia decomposition. Among them, the initial ammonia concentration was found to be the most effective parameter, which is helpful for future research on ammonia decomposition. Based on their analysis of variance results, the significance order of the independent parameters was as follows: initial ammonia concentration > catalyst dosage > solution pH. In addition, the fabrication of ZnO-based heterojunctions is considered to be an effective approach to improve the photocatalytic activity of ZnO. The interface contact of heterojunctions can greatly affect the electron transfer capacity between ZnO and other materials. Mohammadi *et al.*^44^ immobilized TiO_2_/ZnO on a light-expanded clay aggregate (LECA) support to build heterostructures for removal of ammonia from synthetic wastewater. The capability of ammonia decomposition was significantly affected by the mobility and lifetime of the charge carriers generated in the TiO_2_/ZnO composite. Coupling of TiO_2_ : ZnO with a molar ratio of 1 : 2 into a heterojunction photocatalyst showed the lowest photoluminescence emission intensity and maximum photocatalytic degradation activity of ammonia. The optimal pH, catalyst loading, and initial concentration of ammonia were found to be 11 g L^−1^, 25 g L^−1^, and 400 mg L^−1^, respectively. Also, 95.2% ammonia removal was achieved during 3 h of UV irradiation in these optimal conditions. More efforts will need to be focused on the improvement of the exploration of optimal experimental parameters.

## Graphene-based photocatalysts

Graphene is a typical 2D material with a single atomic layer thickness composed of carbon atoms. Graphene is a zero band-gap material with dense honeycomb structures because its valence and conduction bands meet at the Dirac point.^85^ The low Fermi level of graphene also endows it with excellent electrical conductivity as a supporting material. Due to its high electron mobility and special optical, electrical, and mechanical properties, graphene has received extensive attention recently.^86^ When used for photocatalysis, graphene is often combined with other semiconductor materials because it cannot be directly utilized as a photoactive material because of its lack of a suitable bandgap.^87–89^ Graphene provides active sites, charge transfer tunnels or electron enrichment areas to increase the capture of visible light and promote photogenerated electron–hole pair transfer and separation. More active sites on the surface of photocatalysts, higher capacity and efficiency of decomposition will be obtained.

Ammonia decomposition requires much less energy input to occur compared with water splitting; ammonia can spontaneously react with O_2_ to form N_2_O and gives off a large quantity of heat, which provides the energy for water splitting. N_2_O decomposition occurs synchronously under light irradiation. Therefore, ammonia may be able to produce hydrogen by using oxygen to drive the decomposition reaction of water, and the self-modification of nitrogen of the catalyst is beneficial to the production of H_2_. The individual layers of the nitrogen-doped TiO_2_ nanowires (NWs)-intercalated reduced graphene oxide (rGO) membrane are distinguishable, and the TiO_2_ NWs appear to be uniformly distributed between single rGO sheets.^48^ These nitrogen-doped rGO/TiO_2_ NWs hybrids were immersed in ammonia solution, and improved efficiency of hydrogen production from ammonia decomposition under ultraviolet irradiation was observed because rGO inhibited the recombination of the photogenerated carriers in TiO_2_. An interesting phenomenon was demonstrated: the yield of hydrogen production of these nitrogen-doped rGO/TiO_2_ NWs photocatalysts was 14 times higher than that of TiO_2_-P25 and 30-fold higher than that of the TiO_2_ NWs alone under the same conditions, which is probably due to nitrogen self-doping in rGO and the TiO_2_ NWs during the catalytic reaction.

**Fig. 8:**
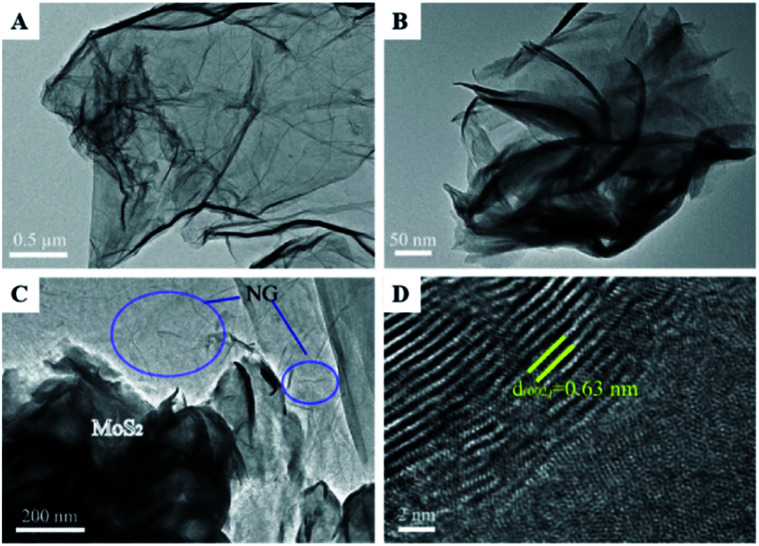
TEM images of the samples (A–D): (A), N-doped graphene (NG); (B), MoS_2_; (C), MoS_2_/NG; (D) high-resolution image of MoS_2_/NG. Reprinted from ref. 47. Copyright 2020, with permission from Elsevier.

**Fig. 9:**
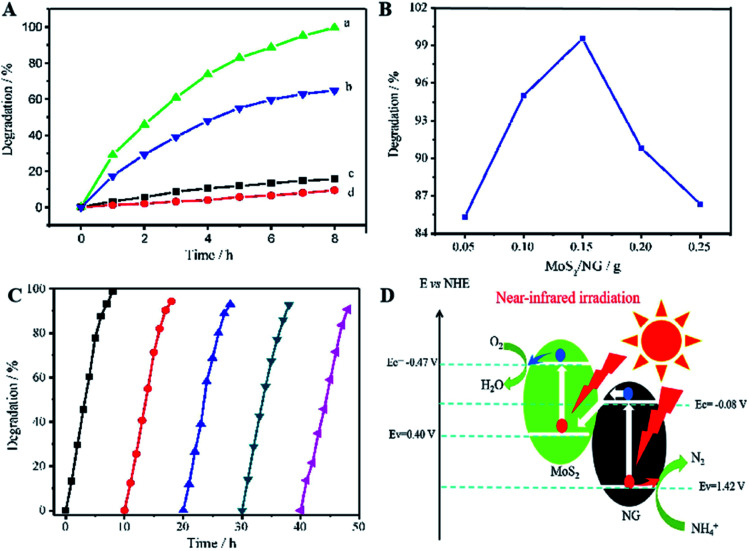
(A) Photocatalytic degradation of ammonia in 50 mL solutions containing NH_3_–N at 100 mg L^−1^ with pH 9.5. (a) 0.15 g of MoS_2_/NG catalyst under NIR irradiation; (b) 0.15 g of MoS_2_ catalyst under NIR irradiation; (c) 0.15 g of MoS_2_/NG in the dark and (d) under NIR irradiation without catalyst. (B) Effects of NG percentage on the ammonia degradation in 50 mL solutions containing 100 mg L^−1^ of NH_3_–N with pH 9.5 under NIR irradiation. (C) Five cyclic uses of the MoS_2_/NG catalyst to verify the stability. (D) The direct Z-scheme mechanism for ammonia degradation *via* the MoS_2_/NG photocatalyst under NIR irradiation. Reprinted from ref. 47. Copyright 2020, with permission from Elsevier.

Similar to this, Zhang *et al.* prepared a photocatalyst consisting of MoS_2_ and N-doped graphene (NG); this heterojunction responds to near-infrared (NIR) light, as shown in Fig. 8.^47^ MoS_2_ is a 2D graphene-like material with a similar layered structure to that of graphene. The bandgap of multilayer MoS_2_ is within 1.29 to 1.8 eV and is even as low as 1.0 eV depending on the layer number and defects, corresponding to 1240 nm NIR radiation; this indicates its possible full use of the NIR irradiation from the solar spectrum. A direct Z-scheme NIR-response photocatalytic system (MoS_2_/NG) was composed of two narrow band-gap MoS_2_ and NG semiconductors to harvest NIR irradiation and degrade ammonia under NIR light irradiation. As shown in Fig. 9A, the ammonia degradation efficiency of the MoS_2_/NG photocatalytic system under NIR irradiation could reach 99.6%, whereas the removal ratio of ammonia was only 64.0% when single MoS_2_ was used as a photocatalyst under similar conditions. This low degradation efficiency was attributed to the low valence band level of the narrow band gap MoS_2_, which was only equal to 0.40 eV, resulting in weak oxidisation. The degradation efficiency increased with the NG content in the initial stage from 1.0% to 5.0% at 8 h. Then, it reached a maximum value of 99.0% when the NG content was 5.0%, as presented in Fig. 9B, resulting from the effective separation of the photogenerated electron–hole pairs; this enhanced the oxidisation ability of the photocatalytic system *via* the combination of MoS_2_ with NG. Moreover, the overload of NG resulted in an electric short circuit, which resulted in decreased degradation efficiency. It should be noted here that the MoS_2_/NG catalyst is highly stable, with an average degradation efficiency of ammonia of over 90% (Fig. 9C) after the fifth cycle. As schematically shown in Fig. 9D, the valence band level (1.42 V) of the composite catalyst was higher than the potential level (−0.276 V *vs.* NHE) of *E*_0_(N_2_/NH_4_^+^), and the composite catalyst could oxidise NH_4_^+^ to N_2_ more effectively. At the same time, the more positive valence band level of the Z-scheme system mainly contributed to the enhanced photocatalytic activity for ammonia degradation.

## Graphitic-C_3_N_4_-based photocatalysts

Graphitic carbon nitride (g-C_3_N_4_), which is only composed of carbon and nitrogen, has been reported to be an attractive, inexpensive metal-free photocatalyst for hydrogen production from water splitting because its reduction and oxidation levels are both located inside the bandgap.^29,90,91^ In addition, g-C_3_N_4_ is fascinating because of its capability for potential applications, including the oxygen reduction reaction, selective organic synthesis, and organic pollutant degradation.^29,92,93^ A new type of atomic single layer graphitic-C_3_N_4_ (SL g-C_3_N_4_) has appealing applications as an inexpensive metal-free photocatalyst in aqueous ammonia treatment.^35^ SL g-C_3_N_4_ in alkaline solution demonstrated higher photocatalytic activity than in neutral or acidic solutions. Under the irradiation of a xenon lamp (195 mW cm^−2^), the TAN (initial concentration 1.50 mg L^−1^) removal rate in the presence of monolayer g-C_3_N_4_ within 6 h reached 80%. The investigation suggested that both photogenerated holes and hydroxyl radicals are involved in the TAN photocatalytic oxidation process and that the major oxidation product is NO_3_^−^–N. In addition, SL g-C_3_N_4_ exhibited good photocatalytic stability in aqueous solution.

**Fig. 10:**
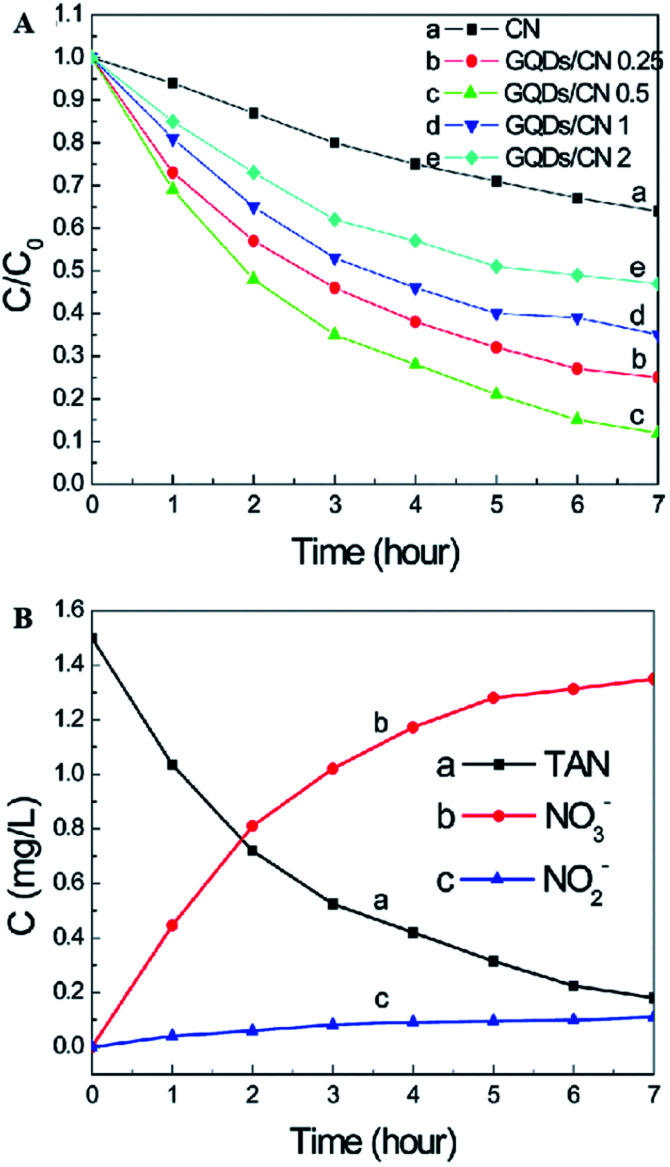
(A) Effects of the g-C_3_N_4_ and GQDs/CN composite dosage on the photocatalytic treatment of TAN solution. (B) Concentration changes of TAN, NO_2_^−^ and NO_3_^−^. Reproduced from ref. 49 with permission from the Royal Society of Chemistry.

A graphene quantum dot (GQD)-modified g-C_3_N_4_ (GQDs/CN) heterogeneous photocatalyst demonstrated degradation properties for ammonia gas.^49^ Through GQD modification, the photon adsorption and electron transfer capability of the heterojunctions were effectively improved, leading to enhancing photocatalytic performance. As clearly shown in Fig. 10, the photocatalyst with 0.5 wt% GQD exhibited the best photocatalytic degradation performance of total ammonia nitrogen, which is approximately 3 times higher than that of pure g-C_3_N_4_. The increased photocatalytic properties can be attributed to the improvement of the photon adsorption ability and electron transfer capacity resulting from GQD modification. It should be noted here that a higher oxygen concentration and pH value are more favorable for the photocatalyst to degrade ammonia to produce NO_3_^−^ (compared with NO_2_^−^).

## Other C-based photocatalysts

In addition to graphene, other carbon-based materials (such as carbon frameworks, carbon nanotubes, and carbon nanobelts) have good electrical and optical properties that can be utilized by coupling the materials with other metal/non-metal materials to build heterogeneous photocatalysts. These heterogeneous structures show improved visible light absorption capacity, surface adsorption capacity, charge transmission, and separation capacities.^45,46,94^ Carbon-based composite photocatalysts (including activated carbon frameworks, carbon nanotubes, and carbon nanobelts) have been reported for the decomposition of ammonia or hydrogen production in the literature.^45,46,94^

Ultrafine TiO_2_ encapsulated in a nitrogen-doped porous carbon framework was fabricated and used as a hierarchical-structured composite photocatalyst to degrade ammonia gas.^45^ After high-temperature calcination, the specific surface area and pore volume of the catalyst increased significantly, which may prompt pollutant molecules to penetrate into the holes and provide more active sites for photocatalysis. The as-prepared ultrafine TiO_2_ encapsulated in the nitrogen-doped porous carbon framework displayed excellent photocatalytic activity and degraded ammonia gas with 100% efficiency after only 5 min of light irradiation. This outstanding performance is related to the small size of TiO_2_, abundant porosity of the composite, and excellent light adsorption. In addition, the nitrogen-doped porous carbon framework in the composite efficiently accelerated the separation of photo-induced electrons and holes.

**Fig. 11:**
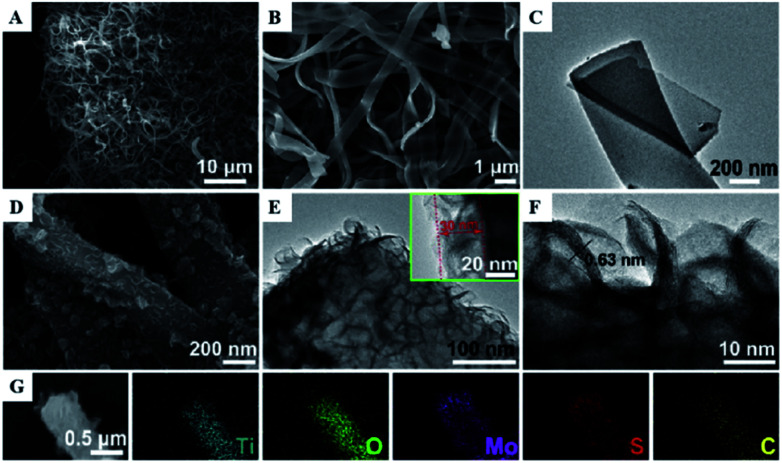
(A) Low, (B) high-resolution FESEM, and (C) TEM image of TiO_2_ CNBs. (D) FESEM, (E) TEM, and, (F) HRTEM images of MoS_2_@TiO_2_ CNBs. (G) EDS elemental maps of Ti, O, Mo, S, and C, respectively. Reprinted from ref. 46. Copyright 2017, with permission from Elsevier.

One-dimensional coaxial nanobelts of molybdenum disulfide grown on titanium dioxide-encapsulated carbon nanobelts (MoS_2_@TiO_2_ CNBs) by electrospinning followed by a hydrothermal reaction method were also prepared and investigated for the photocatalytic degradation of ammonia gas.^46^ The existence of Ti, O, Mo, S and C elements in the prepared TiO_2_ CNBs was confirmed by XRD, and the surface characteristics were observed by FESEM, HRTEM, TEM and EDS (Fig. 10). Under ultraviolet radiation, the MoS_2_@TiO_2_ CNBs heterojunction generates electron–hole pairs and participates in ammonia degradation (Fig. 11). The MoS_2_@TiO_2_ CNBs present excellent photocatalytic activity with an ammonia gas degradation rate of over 91% after 7 min due to the synergistic effect of the robust banding structure and chemical composition.

## Conclusions and outlook

Photocatalytic ammonia decomposition is a prospective technology for future industrial applications for the removal of ammonia and its conversion into harmless H_2_ and N_2_. It appears to be necessary to design heterogeneous structures combining metals or different semiconductors because many bare photocatalysts suffer from one or several limitations, such as poor activity, low stability, low surface area, complicated synthesis approaches, and larger band-gaps. Over the past five years, some significant strategies, such as the metal loading or heterojunction building listed above, have been successfully designed and applied in photocatalytic ammonia decomposition. Despite these significant advances, the photocatalytic efficiency is still too low for practical industrial applications. To address this challenge, future research opportunities and efforts are required in the following aspects:

(1) More effort should be focused on building controllable heterogeneous structures by direct growth on base materials in future studies, which may significantly deepen our understanding of the photocatalytic enhancement mechanisms of ammonia decomposition. It is known that heterojunctions improve the activity to some degree in general; further enhancements will be obtained by controlling their morphologies or nanostructures. The designed morphologies or nanostructures show greater improvements (especially in charge separation and charge transfer capabilities between interfaces) than regular heterogeneous structures.

(2) Nitrogen oxides are easily generated during the photocatalytic decomposition of ammonia owing to the similar redox potentials of these nitrogen-containing products. Although current research has made great progress in the efficiency of photocatalytic decomposition of ammonia, much more research should be focused on controlling the decomposition products and improving the yield of hydrogen, which requires adjustment of the decomposition paths of ammonia to hydrogen by the design of new Z-scheme heterogeneous structures.

(3) Few available photocatalytic materials have been used for photocatalytic ammonia decomposition. The photocatalytic ammonia decomposition performance of more photocatalysts must be investigated in the future. We hope that the present review will stimulate scientific interest in photocatalytic ammonia decomposition using heterogeneous nanostructures as well as in other advanced environmental and energy-related photocatalytic applications.

## Conflicts of interest

There are no conflicts to declare.

## Supplementary Material
